# Stargardt's Connected Research Network Inaugural Meeting: Landscape Review and Horizon Scanning of Stargardt Disease

**DOI:** 10.1167/tvst.14.9.26

**Published:** 2025-09-17

**Authors:** Alexis Ceecee Britten-Jones, Saoud Al-Khuzaei, Matteo Rizzi, Michael D. Crossland, Malgorzata B. Rozanowska, Bernardo S. Mendes, Thales A. C. de Guimaraes, Malena Daich Varela, Dayyanah Sumodhee, William A. Woof, Seemi Khan, Aniz Girach, Susan M. Downes, Michael E. Cheetham, Davide Piccolo, Lauren N. Ayton, Marina Leite Brandão, Hendrick P. N. Scholl, Andi Skilton, Bhavna Tailor, Michel Michaelides, Nikolas Pontikos, on behalf of Stargardt's Connected

**Affiliations:** 1 Department of Optometry and Vision Sciences, Faculty of Medicine, Dentistry and Health Sciences, University of Melbourne, Parkville, Australia; 2 Centre for Eye Research Australia, Royal Victorian Eye and Ear Hospital, Melbourne, Australia; 3 Oxford Eye Hospital, John Radcliffe Hospital, Oxford, UK; 4 Nuffield Laboratory of Ophthalmology, Nuffield Department of Clinical Neuroscience, University of Oxford, Level 6 John Radcliffe Hospital, Oxford, UK; 5 University College London Institute of Ophthalmology, London, UK; 6 Moorfields Eye Hospital NHS Foundation Trust, London, UK; 7 School of Optometry and Vision Sciences, Cardiff University, Cardiff, Wales; 8 Department of Ophthalmology, Faculdade São Leopoldo Mandic, Campinas, São Paulo, Brazil; 9 Centro Universitário de Jaguariúna (UniFAJ), São Paulo, Brazil; 10 Centro Universitário Max-Planck (UniMAX), São Paulo, Brazil; 11 Alkeus Pharmaceuticals, Inc., Davis Square, MA, USA; 12 SpliceBio, Barcelona Science Park, Barcelona, Spain; 13 Stargardt's Connected, Waltham Cross, UK; 14 Belite Bio Inc, San Diego, CA, USA; 15 Medical University of Vienna, Department of Clinical Pharmacology, Vienna, Austria; 16 Pallas Kliniken AG, Pallas Klinik Zürich, Zürich, Switzerland; 17 European Vision Institute, Basel, Switzerland

**Keywords:** Stargardt disease, ABCA4, retinal dystrophy, inherited retinal disease, gene therapy, retinal degeneration

## Abstract

**Purpose:**

The purpose of this study was to update the recent progress in the diagnosis, management, and treatments for Stargardt disease.

**Methods:**

On November 22, 2024, Stargardt's Connected held its inaugural meeting of their Research Network, attended by clinicians, researchers, industry partners, and patient representatives. This meeting aimed to provide an update on Stargardt disease management and research and develop a call to action for the wider community. The format was rapid-fire presentations followed by a roundtable discussion. This review presents the meeting proceeding, along with a summary of best up-to-date evidence and key calls to action.

**Results:**

Topics included: (1) advances in the understanding of Stargardt disease: natural history, genetic basis, animal models, and emerging therapies; (2) supporting individuals with Stargardt disease: disease impact, technological advancements, and lifestyle modifications; and (3) advancing research through stakeholder engagement, research registries, and patient input. The network acknowledges the importance of collaboration among patients, clinicians, researchers, and industry to address critical gaps in the diagnosis, management, and treatment of Stargardt disease. Patient engagement was emphasized as being crucial for driving progress in the field.

**Conclusions:**

Recent years have seen significant progress in understanding and managing Stargardt disease. Key calls to action included the areas of improving genetic testing and counseling, advancing research registries, supporting research and clinical trials, growing multidisciplinary care, addressing lifestyle and dietary modifications, and enhancing technological support.

**Translational Relevance:**

This Stargardt's Connected Research Network initiative outlines a multistakeholder engagement model to discuss ongoing research and emerging treatments for Stargardt disease, a condition with promising therapeutic developments.

## Introduction

Stargardt disease is a rare, inherited retinal disease and the most common form of inherited macular dystrophy.^1^^,^^2^ Early symptoms include progressive central vision loss, difficulty with color vision, photophobia, and difficulty adjusting to the light.^3^ Stargardt disease is most commonly diagnosed in children and young adults, but onset can range from childhood to adults in their 80s.^4^^,^^5^ Although there are currently no approved treatments for Stargardt disease, ongoing research, growing multidisciplinary support, and new adaptive technologies aim to improve quality of life for those living with Stargardt disease. Networks like Stargardt’s Connected offer information for affected families, research updates, and foster community support, driving essential research forward.

Stargardt's Connected held its inaugural meeting of its new Stargardt's Research Network on November 22, 2024. The meeting was attended by leading clinical and research experts in Stargardt disease and patient representatives. The aim of the meeting was to discuss advances and priorities for Stargardt disease care and research, and to develop a call to action for the wider Stargardt research community.

Specific objectives included to share insights and perspectives on what is known about Stargardt disease; discuss treatment modalities being investigated for *ABCA4*-related inherited retinal diseases (IRDs), as this is where the majority of research is currently focused, and the challenges in their development; and identify patient perspectives on the impact of Stargardt disease and discuss support needs. The meeting also aimed to generate a list of priorities and an opportunity to encourage progress in diagnosis, management, and cure through cooperation and shared endeavors.

The format of the network meeting was rapid-fire presentations by both industry and academic researchers followed by a roundtable discussion. This review presents the proceedings of topics discussed at the conference, along with a summary of best up-to-date evidence on these topics, relating to the diagnosis, treatment, and management of Stargardt disease.

## About Stargardt's Connected

Stargardt's Connected has a mission to “raise awareness, give support, and seek a cure.” Founded in 2017 by patients, family members, and friends, and registered as a Charitable Incorporated Organization (CIO) in 2019 (#1183570), Stargardt's Connected aims to address the lack of support and awareness regarding the condition, working with people living with Stargardt disease and their families, and other charities, and support providers, clinicians and researchers, and pharmaceutical and technology companies to deliver its mission. Stargardt's Connected now has over 1000 members around the world (https://stargardtsconnected.org.uk).

As the only registered charity dedicated to Stargardt disease worldwide, Stargardt's Connected is increasingly a “go to” for researchers and industry looking to engage with and recruit patients to research, and for the patients and families looking for information on the latest research and opportunities to participate in studies; particularly those newly diagnosed. Optimistically, whereas a first intervention for Stargardt disease may only be another 5 to 10 years away, it is unlikely that these first treatment options will be universally available, influenced by specific genotype, age, disease state, cost of the intervention, country-specific regulatory and legal restrictions, and availability of appropriately trained clinicians to administer them. That is why it is imperative for Stargardt's Connected to be part of the research conversation to have access to the latest information from research in the field, to support informed access for eligible patients to ethical experimental interventions through registered studies, to share up-to-date information widely in patient-friendly format and language, and to continue to advocate for and support research into mental health and lifestyle interventions more broadly. This was the motivation behind starting the Research Network.

## Stargardt's Connected Community Register

Toward these aims and in addition to acting as advisors to research studies and organizations and funding research, Stargardt’s Connected offers a community register where individuals with Stargardt disease can self-register, aiming to connect the Stargardt community and support research efforts into the disease. Between October 2021 and November 2024, the Stargardt's Connected community register has 356 participants (58% female participants and 42% male participants). The majority of participants registered themselves (*n* = 211), whereas the remaining registered on behalf of a child (*n* = 86) or an adult (*n* = 59) with Stargardt disease.

**Figure 1.**
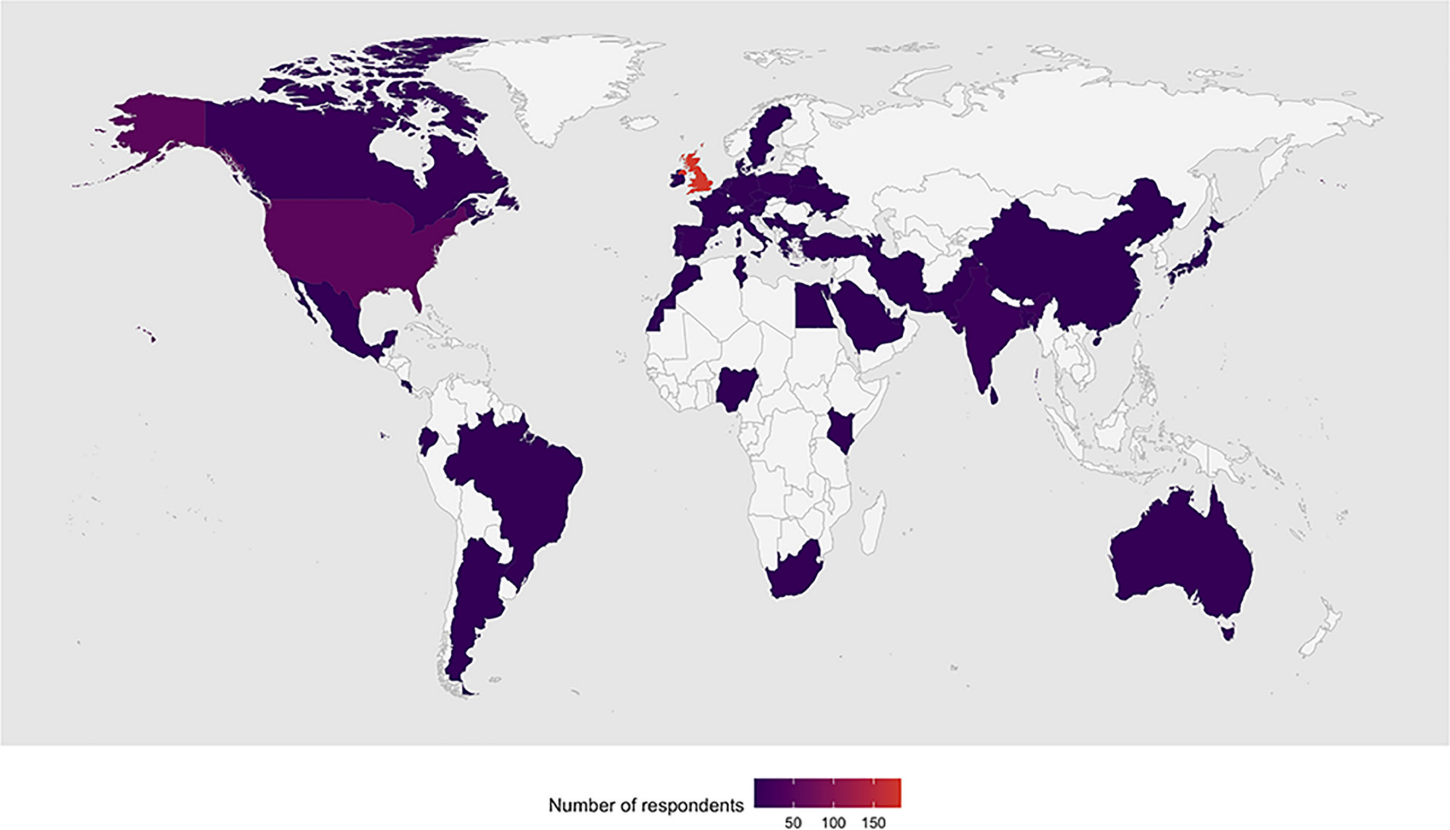
Countries with registrants from the Stargardt's Connected Community Register. A total of 356 registrants from 56 different countries are represented.

Participants originated from 56 different countries (Fig. 1), with approximately half residing in the United Kingdom (52%), followed by the United States (14%), India (4%), and Canada (3%) as the other most common countries of residence. Most (94%) participants self-reported as having a White background. Over 72% (*n* = 255) of the registrants reported having had genetic testing.

Through the register, Stargardt's Connected seeks to build a profile of people living with Stargardt disease around the world that not only provides useful research data but can act as a future pathway for access to research study participation and create the opportunity to grow patient peer networks to overcome geographic isolation.

## Understanding Stargardt Disease: Current Research Landscape

### Epidemiology

Stargardt disease is the most common inherited macular dystrophy.^1^ It is a recessively inherited condition caused by biallelic likely pathogenic or pathogenic variants in the *ABCA4* gene.

Autosomal dominant Stargardt-like phenotypes, associated with variants in *ELOVL4* and *PROM1* genes, have also been described. This present article will concentrate on the current state of research in *ABCA4*-associated Stargardt disease as this is where the majority of research is currently focused. Nevertheless, the scientific community advocates for expanded research, improved understanding, and treatment development across all forms of Stargardt-related macular diseases.

Historically, a prevalence of 1 in 8000 to 10,000 for Stargardt disease has often been quoted in the literature, based on an empirical estimate before the *ABCA4* gene was identified.^6^ However, the actual prevalence of Stargardt disease in different populations has not been established, and is hard to estimate due to clinical heterogeneity, variable age of onset, and incomplete genetic data.^7^ A discrepancy between the genetic prevalence of *ABCA4* variants and the observed phenotypic prevalence of Stargardt disease has been noted.^8^ By computing genetic data from 6 major world populations in global mutation databases, Hanany et al. estimated the proportion of individuals in the population who are expected to be affected with Stargardt disease to be approximately 1 in 6578 individuals, with the highest prevalence in the European population.^9^ However, because some *ABCA4* variants are not always disease-causing, the actual observed prevalence of Stargardt disease may be lower than the estimate from this study.

Based on medical records from referral centers, a study in the Netherlands found a point prevalence of Stargardt disease in 2018 to be approximately 1 in 19,000 to 22,000 individuals, with an annual incidence of 1.67 to 1.95 in 1,000,000.^4^ This estimate is higher than epidemiological data from the United Kingdom, which reported an annual incidence of newly diagnosed Stargardt patients between 1.1 and 1.28 per 100,000 individuals, calculated based on incident cases reported to the British Ophthalmological Surveillance Unit between 2012 and 2013.^3^

Variations in epidemiological estimates could arise from differences in study populations, but are also influenced by study methodologies, disease definitions, and diagnostic criteria. Furthermore, there is an increasing recognition of late-onset Stargardt disease, contributing to a higher proportion of diagnoses in recent years.^10^ Further research toward gaining a better understanding of the actual prevalence of Stargardt disease, especially in developing countries and regions where data is limited and in non-Caucasian populations, can assist in guiding research priorities and developing targeted interventions for this condition. Despite the various epidemiological data reported, *ABCA4* has been identified as the most common causative gene for IRDs in the United Kingdom, United States, and Australia.^1^^,^^2^^,^^11^^,^^12^

### Clinical Presentation and Natural History

There has been significant research carried out to characterize the variability seen in the condition between patients, linking the genetic variants to the appearance of disease in patients, and characterizing the progression of the disease.^7^^,^^13^^,^^14^

**Figure 2.**
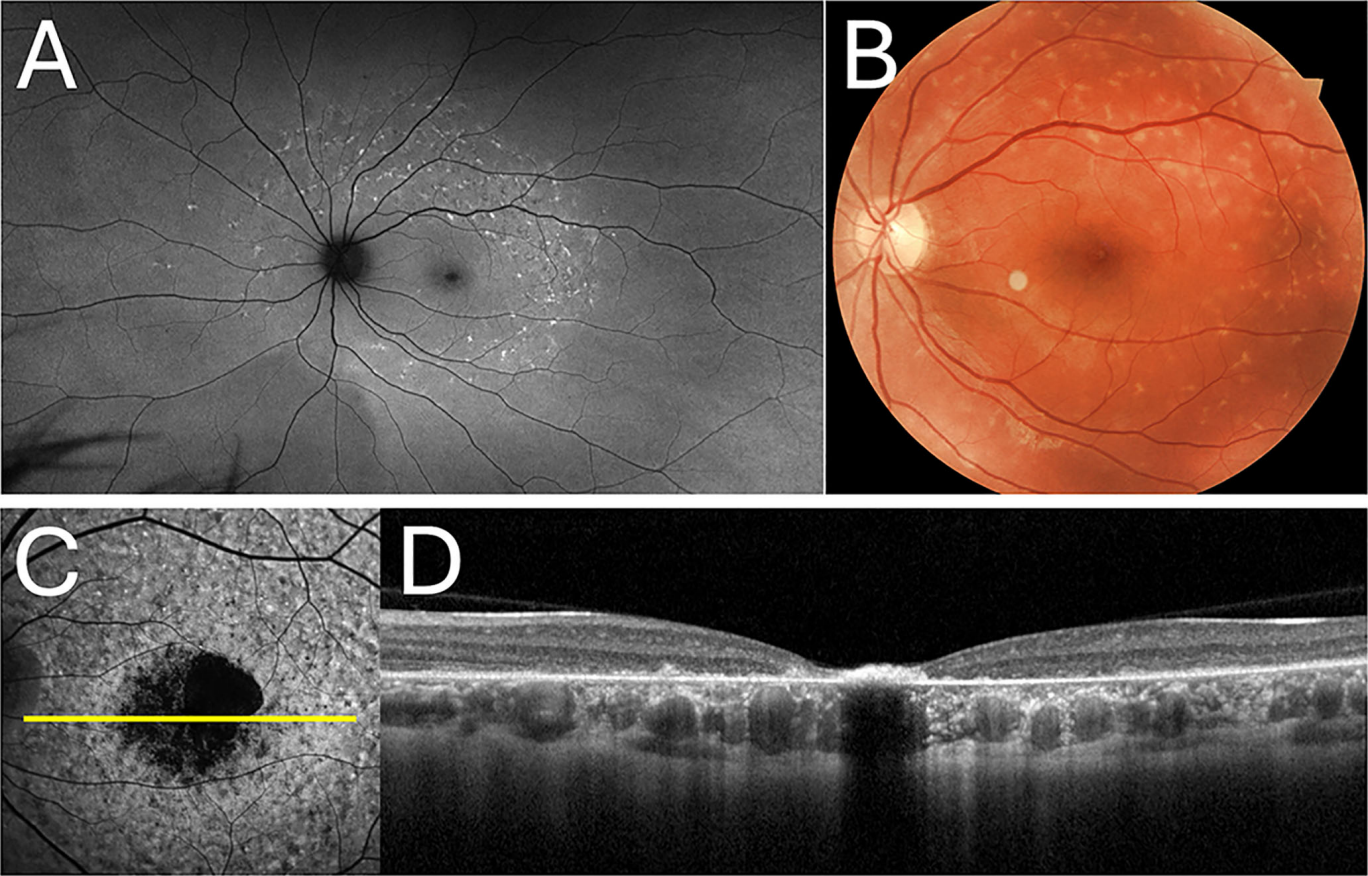
Multimodal images of the left eyes in two individuals with Stargardt disease. (**A**) Ultra-widefield green fundus autofluorescence (FAF) and (**B**) colored fundus images from an individual with mild Stargardt disease. (**C**) The 488 nm FAF and (**D**) optical coherence tomography scans in Stargardt disease captured using the Heidelberg Spectralis.

Stargardt disease (STGD1, MIM #248200) is characterized by the presence of flecks at the early stage, followed by macular atrophy, and peripapillary sparing (Fig. 2). Although symptoms often appear in the first or second decade of life, onset can range from childhood to adults in their 80s, with significant phenotypic variability.^13^ *ABCA4*-related retinopathies also include a broader range of phenotypes, including fundus flavimaculatus, pattern dystrophy, and cone-rod dystrophy. There are typically three phenotypic groups in Stargardt disease, depending on which cells are affected in the retina, assessed with electrophysiology: in type 1, the disease is restricted to the macula; in type 2, the overall cone responses are affected but the rods are preserved; and in type 3, both cones and rods are misfunctioning, in a cone-rod pattern.^5^ This classification has also been correlated with the extension of the areas of atrophy in the posterior pole.^15^^,^^16^

Currently, the largest natural history study of the condition is the multicenter ProgStar study,^17^ in addition to other large cohort studies reported in the literature.^18^^–^^22^ The ProgStar study included 365 individuals (including 251 in the retrospective study and 259 in the prospective study) aged 6 years or older with at least one pathogenic or likely pathogenic variant in *ABCA4*. Eligible participants had at least 1 well-demarcated area of atrophy with a diameter of 300 µm, and a total area of all atrophic lesions of 12 mm^2^ or less at baseline.^17^ Outcome measures included the yearly progression in visual acuity,^23^ retinal structural characteristics on fundus autofluorescence (FAF) and optical coherence tomography (OCT) imaging,^24^^,^^25^ and retinal sensitivity measured using microperimetry. The retrospective arm of the ProgStar study collected data between 2008 and 2014 and the prospective arm consisted of 24 months of observation periods between 2013 and 2017.^26^

Findings from the ProgStar study have been instrumental in characterizing the appearance of macular changes in Stargardt disease, its progression, validation of variants’ pathogenicity, and toward relating the disease severity to the underlying genetic cause. The overall estimated progression of definitely decreased autofluorescence (DDAF; the primary outcome) was 0.74 mm^2^/year (95% confidence interval = 0.64–0.85 mm^2^/year).^25^ Reduction in lesion growth rate has been accepted as a surrogate endpoint by regulatory authorities.^26^

The rate of disease progression in Stargardt disease varies depending on the underlying genetic variants. Recently, the Allikmets group has described a genotype-phenotype correlation matrix, which could be valuable toward providing more tailored prognostic information to patients.^27^ Moreover, the Cremer's group have also devised a method to predict the recurrence risk in offspring based on the variants’ severity, thus paving the way for personalized genetic counselling for Stargardt disease.^28^ Having a genetic diagnosis is also critical for confirming eligibility for emerging gene targeted treatments.

### Genetic Basis of ABCA4-Related Stargardt Disease

Certain genetic variants in the *ABCA4* gene were identified as responsible for Stargardt disease in 1997 by Allikmets et al.^29^ *ABCA4*, located on chromosome 1, is a large gene, comprising 50 exons, with an open reading frame of 6819 base pairs and a total genomic length of approximately 140 kilobases. The high resolution structure of the ATP-binding cassette sub-family A (ABC1) member 4 protein (ABCA4) has been recently described in more detail,^30^^–^^32^ providing insights into its function and supporting studies investigating the expression and function of disease-causing variants in this gene.

Stargardt disease is caused by inheriting biallelic pathogenic variants in the *ABCA4* gene. However, 2 causal variants are only found in 65% to 79% of individuals diagnosed with Stargardt disease, depending on the center,^33^^–^^35^ reflecting the challenges of genetic testing, variant interpretation, and the complexity of the disease.

Recent work aiming at improving our ability to diagnose this condition has identified a number of deep intronic, noncoding splice, structural, and recurrent hypomorphic variants.^36^^–^^39^ Moreover, there are over 4000 reported variants in *ABCA4* listed in ClinVar to date, many of which are variants of unknown significance.^7^^,^^14^ Pathogenicity assessment often requires targeted assays, as well as functional studies like RNA analysis and splicing assays, to accurately detect and characterize the pathogenicity in *ABCA4*-related diseases. Functional studies play a pivotal role in informing the impact of genetic variants on protein function. These studies use biochemical assays to evaluate how specific variants impact the molecular properties of *ABCA4*, including transcription, ATPase activity, and substrate binding (N-Ret-PE).^40^

### Variant Interpretation

Interpretation of *ABCA4* variants is complex. Different rates of progression of Stargardt disease are associated with different combinations of genetic variants. Notably, there is some disagreement in the community about the effect of some of these genetic variants and whether some variants may only be pathogenic if combined with others. There is a global curation effort to evaluate the evidence for each of these genetic variants and standardize the classification of *ABCA4* variants across databases, led by the ClinGen *ABCA4* Variant Curation Panel.^41^

Approaches have been proposed to achieve more consistency in determining the pathogenicity of variants, aligning with established guidelines for genetic variant interpretation.^42^^,^^43^ Cornelis et al. applied an adapted ACMG/AMP classification framework specifically for *ABCA4*,^42^ integrating the point-based system proposed by Tavtigian et al.^44^ alongside ClinGen recommendations.^45^ The proposed system reclassified 1442 of 2246 *ABCA4* variants to pathogenic/likely pathogenic or benign/likely benign, significantly improving our knowledge of previous variants of unknown significance, and providing a valuable resource for clinicians, diagnostic laboratories, and researchers curating variants in *ABCA4*.

However, it is important to note that the ACMG/AMP classification system categorizes a variant as pathogenic if it can cause disease, but does not distinguish variants with reduced penetrance. One of the most well-studied examples is the hypomorphic variant c.5603A>T (p.Asn1868Ile), which is a common variant, with the highest minor allele frequency of 7% in the non-Finnish European population. The c.5603A>T (p.Asn1868Ile) variant is typically classified as a variant of unknown significance, although some laboratories have previously labeled it likely benign or a risk allele. Consequently, it may not be reported by some diagnostic laboratories in clinical reports, and was likely under-reported in earlier studies before 2017 and genetic databases.^42^ This variant was found by Runhart et al. to show approximately 5% penetrance, causing *ABCA4*-related disease, when in *trans* with a deleterious allele^46^; although the rate of penetrance has been argued by Allikmets et al. to be higher.^47^ Phenotypes associated with the c.5603A>T (p.Asn1868Ile) variant are delayed symptom onset and foveal sparing, which at times can be misdiagnosed as age-related macular degeneration (AMD).^48^ Notably, the c.5603A>T (p.Asn1868Ile) variant has also been established to form complex alleles in *cis* with other mild variants, for example, the c.2588G>C (p.Gly863Ala, Gly863del) variant, to cause earlier onset, more severe phenotypes.^42^^,^^48^ Interestingly, the c.2588G>C (p.Gly863Ala, Gly863del) variant, which is classified as pathogenic, does not cause disease by itself and has been found in 50% of complex *ABCA4* alleles.^7^^,^^48^

The ACMG/AMP classification of a variant being pathogenic also does not relate to its severity. Ascertaining genotype-phenotype correlations in *ABCA4* requires knowledge of the severity of *ABCA4* variants on protein function. *ABCA4* retinopathy causes disease on a loss-of-function mechanism.^30^ The clinical phenotype of *ABCA4*-related IRD is influenced by the residual activity of the *ABCA4* protein, resulting from the underlying variants and their combinations.^30^

### Genotype-Phenotype Correlations

Several classification systems have been developed for *ABCA4* according to the proposed severity of the variant,^27^^,^^28^^,^^42^^,^^49^ each serving a distinct purpose. One was developed by the Cremers group based on statistical comparisons of their frequencies in individuals with Stargardt disease versus the general population, informing the variable recurrence risks.^28^^,^^42^ Similarly, a genotype-phenotype correlation matrix was described by the Allikmets group, based on the clinical severity of the associated phenotype, providing insights into disease progression and prognosis.^27^^,^^50^ An F-index had also been described by Molday et al. to classify the consequence of variants on protein function, with F-index from 0 for variants lacking expression and/or activity to 1 for producing the wildtype ABCA4 protein.^30^

There are now also artificial intelligence (AI) approaches for more accurately quantifying phenotypes and biomarkers of retinal degeneration in Stargardt disease such as ellipsoid zone loss and correlating those at scale with genetics data or longitudinally.^51^^,^^52^

Broadly, *ABCA4* variants have been further grouped as null/loss-of-function, severe, moderate, and mild and hypomorphic alleles.^7^^,^^27^^,^^39^^,^^42^ Table 1 shows a summary of the classification in Lee et al.,^27^ presented by Rando Allikmets at the research meeting. However, differentiating between categories can be difficult, as the functional outcome of an allele cannot be determined by variant type. Some nonsense variants do not result in a complete loss-of-function, whereas many missense variants do.^53^

**Table 1.** Summary of *ABCA4* Variant Severity Clarification System as Proposed by Lee et al^27^

Severity Level	Summary of Classification System
Null or loss-of-function variants (PVS1)	Variant is expected to result in null alleles, such as nonsense, frameshifts, canonical ± 1 or 2 splice sites, large multi-exon deletions. These variants skewed toward the most severe clinical phenotypes, and were found to be at a lower-than-expected proportion
Severe	Variants were classified as severe if they were: (1) Observed as the causal allele in trans with hypomorphic alleles (2) Validated by functional studies, where these variants are shown to significantly reduce *ABCA4* expression and basal ATPase activity in HEK293T and patient-derived cell lines (3) Frequently identified in compound heterozygous and/or homozygous state in patients with a poor disease prognosis
Moderate	These variants were proposed to have a partial reduction in expression and basal ATPase activity. Variants were classified as moderate if: (1) There is no intrinsic evidence of severity in their effect on *ABCA4* protein function (2) Functional studies indicate a moderate impact (3) The clinical association is still undetermined
Mild and hypomorphic variants	These variants exhibit expression and functional properties similar to the normal *ABCA4* protein, and were proposed to be associated with a milder prognosis. Hypomorphic variants were proposed to be pathogenic only when paired with a more severe variant on the other chromosome. This class was divided into three groups: (1) The primary disease-causing variant c.5882G>A (p.Gly1961Glu) (2) The common hypomorphic allele c.5603A>T (p.Asn1868Ile) (3) A group of other rare hypomorphic variants, which were associated with mild disease prognosis

### Other Challenges and Considerations

Differences in *ABCA4* allele frequencies have been described between racial and ethnic groups,^8^^,^^54^^–^^56^ requiring careful consideration when interpreting research findings and applying them to different populations. Intrafamilial differences have also been reported among siblings carrying the same *ABCA4* variants.^57^ Further work is needed to investigate unidentified modifying factors influencing phenotypic variability in *ABCA4*-related retinopathies, to improve our understanding of the disease and prognostic accuracy.

Sex has been suggested as a potential variable modifying disease expression in Stargardt disease, as a female predilection was identified among individuals carrying common c.5603A>T (p.Asn1868Ile) and c.5882G>A (p.Gly1961Glu) variants among an international cohort (*n* = 550).^58^ However, Lee et al. did not find an association between sex and mild alleles in a large cohort (*n* = 644) with Stargardt disease from the United States.^59^ *PROM1* and *PRPH2* variants have also been reported to act as modifiers of *ABCA4*-related retinopathy.^60^

Significant progress has been made in recent years, furthering the understanding of *ABCA4* variants and their contribution to Stargardt disease.^7^ The unexplained heritability in undiagnosed patients may be attributed to variants in regulatory regions, deep intronic areas, and rare structural changes. Ongoing research aims to elucidate the pathogenicity and functional impacts of many variants of unknown significance, and explore additional disease modifiers. This work seeks to provide a more comprehensive understanding of the molecular causes and mechanisms underlying Stargardt disease.

#### Stargardt Disease Animal and Cell Models

Research on Stargardt disease relies on available models, including genetically altered (GA) mice (the most common model), zebrafish, and human retinal organoids.

Two main *Abca4* GA mice are available, *Abca4tm1Ght*/J and *Abca4^tm1Kpa^*. In the former, a region spanning the promoter and first exon was replaced, and in the latter, exon 1 was replaced with a *neo* cassette. Both models present a mild phenotype, with fundus autofluorescence increasing over wild-type levels over longer than approximately 6 months. Efforts have been made to develop more severe models. A dual knock-in mouse model was created by introducing 2 homozygous variants in the *Abca4* gene, L541P at exon 12 and A1038V at exon 21, which in humans lead to vision loss starting at the age of 16 years and progresses to complete loss of central vision by the age of 65 years.^53^ However, these mice, like *Abca4*(−/−) knockout mice, accumulate bisretinoid A2E (as evidenced by high-performance liquid chromatography) and lipofuscin-like deposits in the retinal pigment epithelium (RPE; as evidenced by transmission electron microscopy), but do not exhibit photoreceptor degeneration exceeding that of wild-type mice up to the age of at least 12 months.

*Abca4* knockout mice have been crossed with Rdh8 knockout mice.^61^ *RDH8* splicing mutation has been identified in one family with individuals exhibiting retinal features of Stargardt disease.^62^ The *Abca4*(−/−)*Rdh8*(−/−) double knockout mice develop a much more severe phenotype, with significant differences visible at approximately 3 to 6 months. An alternative strategy has involved the exposure of *Abca4* knockout mice to bright light, to induce retinal degeneration. This has exacerbated the model, by causing a reduction in outer nuclear layer thickness (approximately 10–20%).^63^ Typical measures in *Abca4* knockout mice involve fundus autofluorescence, thickness of photoreceptor layer (by histology or OCT), and measures of lipofuscin accumulation in the RPE (e.g. A2E content). Overall, *Abca4* mice present a milder phenotype than the human counterpart. This is potentially also due to the lack of a macular/foveal region in the mouse eye, which is the most affected part of the retina in Stargardt disease. Nonetheless, a mouse lacking *Abca4* can be useful for the testing of gene therapy strategies that aim to restore *Abca4* expression, because it provides a “blank canvas” to measure expression of *Abca4* delivered by gene therapy.

Alternative animal models could be considered. For example, zebrafish possess two *Abca4* orthologs, *Abca4a* and *Abca4b*. Research is ongoing in developing fish lacking either of these and testing whether the transgenic animals recapitulate features of Stargardt disease.^64^^,^^65^

Finally, an alternative to animal models could be the use of retinal “organoids” derived from patients with Stargardt disease. The organoids are retina-like structures derived from patient-derived stem cells (induced pluripotent stem cells). The organoids contain all major neuroretinal cell types and roughly recapitulate development of the embryonic human retina. However, they only survive approximately 1 to 1.5 years and thus do not recapitulate the phenotype of a condition that takes several years to manifest itself. However, they can be useful to study the effect of the disease at the single-cell level (for example, the trafficking of molecules to the cell's outer segment, or the alternative splicing of *ABCA4*). Furthermore, they enable testing genetic therapeutic approaches in human tissue. Overall, current models for Stargardt disease are extremely useful for the testing of therapeutic strategies but do not fully recapitulate all the features of the disease and could therefore be improved to better model the pathophysiology of Stargardt disease.

#### Emerging Therapies

Several recent reviews have explored and discussed emerging therapeutic approaches for Stargardt disease.^14^^,^^66^ Currently, there are no commercially available therapies for Stargardt disease. Broadly, emerging therapies being developed for Stargardt disease include:

(1) Pharmacological therapies, targeting different aspects of the visual cycle in order to reduce the toxic accumulation of retinaldehydes and lipofuscin in the retina, or removal of existing lipofuscin.^67^
(2) Gene therapy, whereby a functional gene product is delivered via a subretinal injection. The size of the *ABCA4* gene is too large to be delivered in a single adeno-associated virus vector. Approaches to overcome this limitation to restore the expression of the full-length *ABCA4* protein include using larger vectors (e.g. lentiviral vectors), splitting the gene and using dual vectors,^68^^,^^69^ and non-viral vectors.
(3) Antisense oligonucleotides (AONs), which aim to promote correct splicing at the pre-mRNA level by blocking recognition of spliceosome binding sites, is currently being investigated for specific splicing variants in *ABCA4*.^70^^,^^71^
(4) Genome editing, such as clustered regularly interspaced short palindromic repeat (CRISPR) based approaches, base editing, epigenetic repression, and prime editing. Currently, 63% of known *ABCA4* variants are thought to be potentially editable.^72^ In addition, RNA editing strategies, using pre-mRNA trans-splicing technology, are also being developed. Base editing has shown promise in mouse models and NHPs^73^; it has not yet progressed to human clinical trials.
(5) Stem cell-based therapies, aiming to replace the diseased RPE cells with RPE made from human embryonic stem cells (hESCs), induced pluripotent stem cells (iPSCs), and bone marrow-derived stem cells.
(6) Optogenetics, which introduces light-sensitive proteins (opsins) into specific retinal cells, for it then to activate light to restore vision in advanced stages of the disease.

Table 2 shows a summary of the clinical trials on Stargardt disease in humans (status updated as at February 10, 2025).^74^^–^^80^

**Table 2.** Treatments for Stargardt Disease Registered on clinicaltrials.gov (February 2025)

Treatment Type	Intervention and Comparator	Study Design	Clinical Trial Information	Sponsor
**Pharmacological therapies targeting toxic bis-retinoids**				
Gildeuretinol	ALK-001, oral administration for 24 mo (Comparator: placebo)	Placebo-controlled RCT	NCT02402660 (TEASE, Phase II, recruitment completed); NCT04239625 (Open label extension)	Alkeus Pharmaceuticals, Inc
Tinlarebant (RBP4 antagonist)	LBS-008, oral administration of 5 mg/day for 24 mo (Comparator: placebo)	Placebo-controlled RCT	NCT06388083 (Phase II/III, recruiting); NCT05244304 (Phase III, recruitment completed)	Belite Bio, Inc
STG-001 (RBP4 antagonist)	STG-001 oral administration, (two dosage groups), for 28 days	Open-label, non-randomized study	NCT04489511 (Phase II, completed 2024, results submitted online)	Stargazer Pharmaceuticals, Inc
Emixustat	Emixustat hydrochloride, ACU-4429, oral administration of 10 mg/day in the Phase III trial (Comparator: placebo)	Placebo-controlled RCT	NCT03033108 (Phase II, completed in 2017);^75^ NCT03772665 (Phase III [SeaSTAR], completed in 2022, results posted online)	Kubota Vision Inc
4-methylpyrazole	4-methylpyrazole (4-MP, fomepizole, Antizol) infusion 15 mg/kg dose (Comparator: saline)	Placebo-controlled RCT	NCT00346853 (Phase I, completed in 2006, no results posted)	University of Utah
**Gene therapy, optogenetics, and RNA therapy**				
RORA adeno-associated virus vector gene therapy	OCU410ST single subretinal injection	Open-Label, non-randomized, dose escalation study (Phase I) and randomized dose expansion study (Phase II)	NCT05956626 (GARDian, Phase I/II, recruiting)	Ocugen
Adeno-associated virus vector gene therapy	JWK006 single subretinal injection	Open-Label, non-randomized, single ascending dose study	NCT06300476 (phase I/II, active, not recruiting)	West China Hospital
Lentivirus (Equine infectious anemia virus (EIAV))	SAR422459 single subretinal injection	Open-Label, non-randomized, single ascending dose study	NCT01367444 (Phase I/II, terminated); NCT01736592 (follow-up study of the 27 participants enrolled)	Sanofi
Optogenetics Therapy	vMCO-010 (1.2E11gc/eye) multi-Characteristic Opsin single intravitreal injection	Open-label, non-randomized trial	NCT05417126 (STARLIGHT, Phase 2, completed 2023, results submitted online)	Nanoscope Therapeutics Inc
RNA exon editor	ACDN-01, single subretinal injection (3 doses)	Open-Label, non-randomized, single ascending dose study	NCT06467344 (STELLAR, Phase I/II, recruiting)	Ascidian Therapeutics, Inc
**Stem cell therapy**				
hESC-derived retinal pigment epithelium cells RPE cells	MA09-hRPE, single subretinal injection (4 dose groups between 50,000–200,000 cells)	Open-Label, non-randomized trial	NCT01345006, NCT01469832 (Phase I/II, completed in 2015);^76^ NCT02445612, NCT02941991 (follow-up study, completed 2019)	Astellas Institute for Regenerative Medicine
hESC-derived retinal pigment epithelium cells	MA09-hRPE single subretinal injection with 50000 cells	Open-Label, non-randomized trial	NCT01625559 (Phase I, completed in 2015)^77^	CHABiotech CO., Ltd
hESC-derived retinal pigment epithelium cells	hESC-RPE, delivered as either a single subretinal injection with 100,000 cells, or subretinal implantation seeded in a monolayer in a polymeric substrate	Open-Label, non-randomized trial	NCT02903576 (Phase I/II, completed in 2019)	Federal University of São Paulo
Bone marrow-derived stem/progenitor cells	Autologous bone marrow derived stem cells (BMSC), delivered as retrobulbar, subtenons, intravitreal or subretinal and intravenous injections	Open-Label, non-randomized trial	NCT01920867 (SCOTS, completed in 2020);^78^ NCT03011541 (SCOTS2, recruiting)	MD Stem Cells
bone marrow-derived stem/progenitor cells	Autologous bone marrow stem/progenitor cell intravitreal injection in enrolled patients by repeated follow-up over 1 y with clinical evaluations	Open-Label, non-randomized trial	NCT03772938 (Phase I, status unknown, no results posted)	Pomeranian Medical University Szczecin
**Other therapies**				
Complement C5 Inhibitor	Avacincaptad pegol (Zimura) intravitreal injection monthly for monthly for up to 17 mo (Comparator: sham injection)	Placebo-controlled RCT	NCT03364153 (Phase II, active, not recruiting)	Astellas Pharma Global Development, Inc
Saffron	Saffron oral supplementation 20 mg/day (Comparator: placebo)	cross-over Placebo-controlled RCT	NCT01278277 (Phase I/II cross-over RCT)^79^	Catholic University of the Sacred Heart
Omega-3 fatty acid	Combined long-chain omega-3 fatty acid (3660 mg/day eicosapentaenoic acid [EPA] and docosahexaenoic acid [DHA]) for 24 weeks (Comparator: placebo (sunflower oil))	Placebo-controlled RCT	NCT03297515 (MADEOS, completed)^80^	Ophthalmos Research and Education Institute
Omega-3 fatty acid (docosahexaenoic acid)	Docosahexaenoic acid oral supplements (2000 mg/day) for 3 mo (Comparator: placebo (corn oil and soy oil))	Cross-over Placebo-controlled RCT	NCT00060749 (Phase I, completed in 2007)^74^	National Eye Institute (NEI)
Metformin	Metformin oral administration of metformin of 500 mg/day at study entry and titrated up to reach 2000 mg/day	Open label, single group treatment	NCT04545736 (Phase I/II, recruiting)	National Institutes of Health Clinical Center (CC)
Disulfiram	Disulfiram (Antabuse) oral administration 250 mg/day	Open label, single group treatment	NCT06319872 (Phase I, recruiting)	University of Rochester
Acupuncture	Acupuncture and massage therapy (initially once/week for 24 mo)	Open-label, single group treatment	NCT02255981 (completed, results posted online)	Escuela Neijing
Microcurrent stimulation therapy	Microcurrent Stimulation Therapy, no further information	Not specified	NCT01790958 (Phase I, completed in 2012, no results posted)	Retina Institute of Hawaii

RCT, randomized controlled trial.

Treatments for Stargardt disease is a rapidly evolving field with several promising approaches. In addition to the clinical trials listed in Table 2, as at January 2025, the United States Food and Drug Administration has authorized the launch of clinical trials for 2 other potential therapies: a protein splice therapy (sponsor = SpliceBio, Inc.) and an mRNA trans-splicing gene therapy (sponsor = ViGeneron) for Stargardt disease. Whereas challenges remain in the need for early intervention and addressing genetic heterogeneity, the diverse approaches that are being developed offer promising prospects for effective therapies for individuals living with Stargardt disease.

A 2007 phase I study of docosahexaenoic acid (DHA) dietary supplementation was initiated (NCT00060749), underpinned by limited evidence that impaired biosynthesis of fatty acids, including DHA which is in high concentration in the retina, may be a contributing factor of Stargardt macular degeneration. However, the study reported no perceived benefit.^74^

## Supporting Individuals With Stargardt Disease

In addition to research efforts focused on developing treatments, the Stargardt's Connected network emphasized the importance of supporting individuals living with Stargardt disease and multidisciplinary care. Recent advances and strategies on supporting individuals living with Stargardt disease are hereby discussed.

### Multidisciplinary Clinical Networks

Multidisciplinary clinical networks are essential for providing integrated care, and growing these networks was proposed as a way to promote research participation. Optometrists and allied healthcare professionals can support diagnosis, provide low vision care and ongoing monitoring, and assist in managing syndromic symptoms. In the United Kingdom, Eye Clinic Liaison Officers (ECLOs) are particularly valuable, as they assist patients after the initial diagnosis by connecting them with relevant healthcare practitioners and support networks outside the clinic. However, the ECLOs are funded through NHS charitable routes, such as the Royal National Institute of Blind People (RNIB), and as such are not universally available.^81^ Genetic counselors and geneticists are critical in providing genetic counseling, and facilitating the understanding and implications of genetic diagnoses. Improving integrated networks can elevate the care for all individuals living with Stargardt disease, reaching patients across all disease stages and locations.

### Schooling and Education

As symptoms of Stargardt disease often emerge during school years, vision impairments often impact schooling and education. Diagnosis can also affect social relationships and influence career choices.^82^^,^^83^ Visual tasks and learning often require increased effort due to scanning magnified text and visual fatigue, with the level of fatigue not always correlating with severity of vision loss.^84^ Visual fatigue can also manifest as headaches, tiredness, or behavioral changes throughout the day.

Children and young people with Stargardt disease often need additional support and adaptation strategies to help meet their needs in the education environment, often provided by a specialist teacher for vision impairment. It is important to consider and regularly reassess tailored accessibility strategies to meet specific needs as workloads increase throughout schooling, and as the disease progresses. Examples of support include enlarged print, relay systems for accessing whiteboard content, frequent rest breaks, additional time to complete tasks, and the use of low vision aids and adapted learning methods such as an increased use of audio-based resources. The Stargardt’s Connected Educational Toolkit offers guidance for schools, parents, and young people managing vision loss in educational settings.^85^

### The Emotional Impact of Stargardt Disease

Although little research has investigated the specific impact of Stargardt disease on mental health and wellbeing, people with other forms of vision impairment have higher rates of depression,^86^ anxiety,^87^ and post-traumatic stress disorder,^88^ as well as lower mental wellbeing^89^ than the general population. Qualitative research with young people with Stargardt disease and their parents indicates frequent emotions such as frustration, irritability and difficulty concentrating.^82^

Psychological interventions, such as counseling,^90^^,^^91^ problem-solving therapy,^92^^–^^96^ positive psychology training,^97^ cognitive behavioral therapy,^98^^–^^101^ and acceptance and commitment therapy,^102^ may improve mental health in people with vision impairment and there is a general view that these approaches should be offered at an earlier stage in the disease process.^103^ Much of this research has focused on older adults and there remains a significant knowledge gap in this area of research,^104^ although two current randomized controlled trials may help to provide evidence for the benefit of two therapies: acceptance and commitment therapy^105^ and cognitive behavioral therapy.^106^

One factor that appears to limit wellbeing in older adults with vision impairment is activity limitation.^107^ Optical and electronic low vision aids, assistive technology, and rehabilitation services can improve task performance,^108^^,^^109^ and may therefore maximize mental wellbeing in people with vision impairment. Referral to a low vision clinic should be offered routinely to people with Stargardt disease. One limiting factor is that the provision of services to people with vision impairment is often fragmented, with support being provided by local education services, hospitals, community optometrists, and third sector organisations.^110^ Integration of these services and the use of a link worker would improve the provision of comprehensive rehabilitation programs to those with Stargardt disease.

Finally, wellbeing may be enhanced through peer support from the many people who live full and rewarding lives with Stargardt disease who can provide positive role models and give practical advice on living with this condition.^111^^–^^113^

### Technology and Vision Aids

Advances in consumer technology have been adopted by many people with vision impairment to improve access to print, to help with navigation, and to describe scenes. The ubiquity of electronic text has largely replaced the need for large print books or newspapers, as people can manipulate text to its most appropriate size, brightness, viewing position, and contrast.^114^

It is now common for people to use smartphone cameras as a magnifier, either by photographing scenes and zooming on the image,^115^ or by use of a third party magnifier app such as Supervision+.^116^^,^^117^ This approach has been shown to be as effective as using a stand-alone video magnifier.^118^ Further, the LED torch on a smartphone can be used to illuminate scenes in dim light.

Wearable electronic low vision aids, comprising a camera and a video display mounted on a headset, have been available since the 1990s.^119^^–^^121^ Some devices, such as eSight, use bespoke hardware,^113^ whereas others use commercially available virtual reality systems.^122^ In either case, software provides an enlarged, high contrast view of the world. Although these systems can provide dramatic improvements in visual acuity,^123^^–^^125^ and contrast sensitivity,^122^^,^^126^^,^^127^ uptake of these devices remains low. In one study, less than half of the 60 people who trialed one of these systems said they would use it in their daily life, with none actually purchasing one.^122^ Even among those who have one of these systems, approximately one in five people stop using their device after a few months. Barriers to using these systems include their physical appearance, their weight, image lag, and short battery life.^121^^,^^128^^,^^129^ as well as users’ motivation and change in vision.^130^ Notably, performance on activities of daily living is not improved systematically with these devices, even when users performed better on clinical tests.^131^

Sensory substitution is a term which relates to using a different sense in place of vision. The most common approach to this is to use audio resources (for example, listening to an audiobook instead of reading in print, or using text-to-speech technology), and speech-to-text (such as dictating a text message using a smart assistant such as Siri or Alexa). Such devices can be used by those with all levels of vision. Additionally, screen reader software, such as Narrator on Windows, VoiceOver on Apple devices, and TalkBack on Android OS, allow accessibility to be offered routinely on nearly all computers, tablets, and smartphones.

AI can be used to read printed text aloud, either by using the camera on a smartphone with an app like SeeingAI,^132^ or on a stand-alone device worn on spectacles such as Orcam.^133^ Studies have shown both approaches to be successful, although there is no significant difference between SeeingAI and Orcam in reading ability.^134^

One of the most exciting developments in technology for people with vision impairment is the increased use of AI to describe scenes.^135^^,^^136^ As well as being available as free-to-download smartphone apps, services like BeMyAI are beginning to be installed on mainstream devices, such as the RayBan/Meta smart glasses.

This is an exciting and rapidly changing field and the interested reader is directed to podcasts, such as “The Blind Life” and BBC's “In Touch.”

### Lifestyle and Dietary Modifications

Tobacco smoking has also been identified as a risk factor that can negatively impact macular function and, in a retrospective study, was associated with poorer visual acuity outcomes in retinitis pigmentosa.^137^ There is also some evidence that lower intake of vitamin A (<600 µg RAE/day from a single cross-sectional study) is associated with better visual acuity outcomes in Stargardt disease.^138^ However, the multicenter prospective Progstar study did not find a higher risk of loss of best-corrected visual acuity to be associated with vitamin A use or smoking over 1 year in Stargardt disease.^139^ Nonetheless, patients are generally recommended to avoid smoking and supplements containing vitamin A based on their contributions to models of Stargardt disease pathogenesis.^140^ Similarly, whereas there is currently no evidence that reducing dietary intake of vitamin A-rich foods will reduce the progression of Stargardt disease, foods containing high vitamin A content (such as liver and fortified foods) and medications containing retinoids are generally advised to be used with caution. The effects of topical vitamin A on Stargardt disease progression has not been evaluated. While Stargardt's Connected has taken steps to bring together what limited data are available on dietary modifications in their “Vitamin A and Stargardt's Disease” booklet, available through their website, it remains an area of significant interest and importance for the Stargardt's community in the absence of therapy. Managing one's diet is something that people can proactively do and take control of in order to try to minimize the impact of the condition and in the absence of a therapeutic option.

Light exposure triggers the hydrolysis of all-trans-retinaldehyde from visual pigments and accelerates accumulation of bis-retinoids, N-retinylidene-N-retinylethanolamine (A2E) in the lipofuscin of the RPE, which can induce oxidative damage and accelerate photoreceptor degeneration in Stargardt disease.^141^^,^^142^ A small study (*n* = 5) found that eyes protected from light showed slower progression of decreased autofluorescence,^143^ and thus wearing protective dark tinted spectacles in bright conditions could reduce the risk of light toxicity. All-trans-retinaldehyde is toxic and its toxicity increases upon exposure to UV, violet, and blue light up to 460 nm that leads to its photoexcitation and the interaction of that excited state with oxygen lead to formation of damaging reactive oxygen species. Therefore, studies are also evaluating the effects of blocking light below 460 nm in protection from light-induced retinal injury in models of Stargardt disease.^144^ Some patients are advised to wear yellow or amber tinted glasses as a potential safeguard, based on the theoretical basis that blocking blue light can protect from damage at molecular level.

## Advancing Research

Stargardt’s Connected highlights the critical role of patient registries and patient involvement in driving clinical trials and research progress. Advancing research in Stargardt disease requires multistakeholder engagement, including affected individuals and their families, international research teams, clinicians, genomic centers, and industry partners, playing key collaborative roles. Patient involvement was emphasized as being critical for understanding disease prevalence and natural history and identifying candidates for future therapies. The discussion highlighted several areas for future advancement, summarized as follows.

### Research Registries

Establishing and growing patient registries and databases was discussed as a way to effectively engage patients, enabling a collection of information from individuals who are interested in being involved in research and clinical trials.

There are several IRD research registries run by research teams, including in Australia,^145^ Portugal,^146^ and across parts of Asia,^147^ and by industry groups (NCT06591806 and NCT06435000). These registries often aim to study IRD prevalence, genetic profiles, genotype–phenotype correlations, and natural history, as well as to assist in the recruitment of participants for emerging treatments/clinical trials. In research registries where data are collected by researchers or clinicians, advantages include better data quality control, standardized data collection methods, and clinical data access. However, they are often more resource-intensive and have potential for selection bias.^145^

Patient self-enrollment registries, for example, the My Retina Tracker Registry (Foundation Fighting Blindness, USA^148^) and Stargardt's Connected Patient register, enables individuals affected to voluntarily enroll themselves and provide their own data through online portals. Limitations of self-enrollment registries include poor data quality or accuracy, incomplete information and missing data, and lack of clinical data or standardization. Self-selection bias can also disproportionately favor participants from well-represented backgrounds and those who are more motivated or engaged in research. However, by allowing patients to directly self-enroll and contribute their data, these registries aim to increase patient engagement, empowering individuals affected to take an active role in research. They are also powerful consumer driven resources demonstrating that there are people in the region who need new treatments.

Patient organization-led registries also allow for patient communities to collect data to address the questions they feel are important to ask. Until recently, sex was not considered a disease modifying-variable in Stargardt disease, and this is now being debated within the literature.^58^^,^^59^^,^^149^ This raises the possibility that there are other disease modifying factors out there which research-led registries may not be collecting the necessary data around, because they have yet to be reported on. Patient-organization-led registries also enable patients to retain control over their data, to have a voice in how these data are used, and what it is used for.^145^

Challenges in sustaining funding was discussed at the research network meeting as a key consideration for establishing and maintaining registries. Assessing participant eligibility often requires researchers or clinicians with disease-specific expertise, especially in a condition like Stargardt disease with nuances in genetics and clinical heterogeneity. Trained personnel are needed for ongoing data collection, recruitment, and monitoring. Systems need to be developed to meet multisite ethics and privacy requirements, and enable data standardization and data quality assurance. Other considerations include aligning with existing clinical and research workflows and processes to minimize the burden on clinicians, researchers, and patients; allowing for scalability as research evolves; and accommodating multiple languages in international registries to promote equitable participation. Balancing the need for high-quality data and active participant engagement is a key consideration in designing and implementing research registries.

### Improving Diagnosis

Having a genetic diagnosis is required for inclusion in gene-specific therapies and some pharmacological therapies (see Table 2); although a clinical diagnosis of Stargardt disease is enough for meeting the eligibility criteria of some gene-agnostic treatment trials. A genetic diagnosis can also inform prognosis,^27^ enable more accurate counseling,^28^ and provides personal utility empowering affected individuals to engage with research.^150^^,^^151^

As discussed earlier, interpreting the genetics of *ABCA4* is complex. Integrated analysis of clinical and genetic data is crucial for proper diagnosis and prognosis, and often requires trained personnel to verify and interpret genetic reports. Limitations in sequencing methods must be considered. Some clinical sequencing (e.g. exome or targeted-panel sequencing of coding variants) may not cover certain regions of the *ABCA4* gene, such as deep intronic regions, or detect large structural variants. These are estimated to account for 12% to 15% of all *ABCA4* variants^7^ and a significant portion of unsolved *ABCA4* cases.^34^^,^^38^ Phasing is important for confirming the disease-causing variants, especially in light of the number of complex alleles and *cis* modifiers that are known in *ABCA4*. In cases where parental samples are not available, long read sequencing is an emerging technique with potential applications.^152^

Globally, there are additional differences in access to genetic testing and care provision.^147^^,^^153^^,^^154^ The United Kingdom has centralized funding for rare disease testing, including for individuals diagnosed with Stargardt disease.^155^ In some countries with limited public funding, implementation of sponsored genetic testing programs has significantly increased access, mainly by subsidizing costs.^145^^,^^148^^,^^156^^–^^158^ However, in regions with no or limited funding and access to genetic testing as part of clinical services, genetic testing for Stargardt disease often relies on academic research programs or industry funding. Addressing equitable participation is important for enhancing our understanding of Stargardt disease and delivering comprehensive care. A global survey on genetic testing experiences of people with IRD done by Retina International (410 respondents over 30 countries) reveals that both genetic testing and genetic counseling services are not equitably accessible.^159^ Furthermore, 37% of respondents answered that their eye care professional was either unaware of a genetic test, remained neutral, or did not encourage them to undergo the test. The survey also shows a long waiting time to undertake the test, with 57% of respondents confirming they had to wait for more than 3 years before obtaining a genetic diagnosis. The study concludes that more training and awareness about genetic testing are required for patients, families, and eye care professionals worldwide. A more recent survey has also highlighted that there remain several key gaps and disparities in the pathway for patients,^160^ and that AI approaches such as Eye2Gene could potentially help address these issues, especially in parts of the world where access to genetic testing is limited.^161^^,^^162^

## Closing Summary

The inaugural meeting of the Stargardt's Connected Research Network has underscored the significant progress made in understanding and addressing Stargardt disease, in particular for *ABCA4*. The collaborative efforts of clinicians, researchers, industry partners, and patient representatives have highlighted several key areas for future advancement. Despite the strides made, there remain critical gaps that need to be addressed to improve diagnosis, management, and treatment of Stargardt disease and associated macular dystrophies.

### Calls to Action

Enhance Genetic Testing and Interpretation:

• Increase access to comprehensive genetic testing globally, ensuring equitable participation across different regions and populations.
• Provide training for eye care professionals on the importance and availability of genetic testing and counseling.
• Develop standardized protocols for interpreting *ABCA4* variants, including deep intronic and structural variants, to enhance diagnostic accuracy.

Advance Research Registries:

• Secure sustainable funding for patient registries to ensure ongoing data collection, recruitment, and monitoring.
• Encourage patient enrollment in registries to increase engagement and empower individuals to contribute to research.
• Promote participation from diverse backgrounds, especially from under-represented groups, to enhance knowledge across different populations and foster more inclusive and equitable research outcomes.
• Implement systems to ensure high-quality data collection and standardization across registries.

Research and Clinical Trials:

• Continue research to address gaps in understanding the natural history, disease progression, and potential disease modifiers for Stargardt disease, as well as the cost-of-illness, the experience, burden, and treatment expectations for those living with Stargardt disease.
• Advocate for increased funding and regulatory support for clinical trials investigating emerging therapies, such as gene therapy, pharmacological treatments, and stem cell-based interventions.
• Partner with industry to help accelerate promising therapeutic candidates from the bench into clinical trials through to registration and approval, and expanding access to trials across regions and populations.
• Encourage patient partnership and involvement in the identification of research topics and design of clinical studies to ensure these address the needs of people living with Stargardt disease.
• Incentivize research to tackle research disparities for those with macular diseases associated with *ELOVL4* and *PROM1* variants. As of April 2025, there are no gene-targeted treatments for *ELOVL4* or *PROM1*-related retinal diseases registered on clinicaltials.gov.

Support Multidisciplinary Care:

• Establish and grow multidisciplinary clinical networks to provide integrated care, including genetic counseling, low vision services, and psychological support.
• Understand the optimal form of mental health and wellbeing support for people with Stargardt disease, and build the evidence base for the development and provision of these services.
• Enhance support services such as ECLOs to assist patients post-diagnosis and connect them with relevant healthcare practitioners and support networks.
• Advocate for individualized and up-to-date accessibility education, health, and care plans to improve the long-term educational and psychosocial outcomes of children and young people with Stargardt disease.

Address Lifestyle and Dietary Modifications:

• Raise awareness about the impact of lifestyle factors such as smoking and vitamin A intake on Stargardt disease progression and promote the use of protective eyewear to mitigate light-induced retinal damage.
• Incentivize more research on lifestyle and dietary modifications, including supplementation, to develop a definitive understanding of their potential role in slowing progression of Stargardt disease.

Enhance Technological Support:

• Encourage the development and adoption of advanced vision aids, assistive technologies, and AI-powered applications to improve the quality of life for individuals with Stargardt disease.
• Advocate for the integration of vision-specific educational strategies that include funding to access assistive technologies and tailored learning resources supported by programs, such as Qualified Teachers of Vision Impairment (QTVIs).

By addressing these gaps and implementing these calls to actions, the Stargardt's Connected Research Network can continue to drive progress in the diagnosis, management, and treatment of Stargardt disease, ultimately improving the lives of those affected by this condition. Stargardt's Connected is keen to hear from any individuals, groups, and organizations who are interested in collaborating as part of the Research Network to help address these priorities.
